# Light-chain proximal tubulopathy: a retrospective study from a single Chinese nephrology referral center

**DOI:** 10.1080/0886022X.2023.2283587

**Published:** 2024-02-19

**Authors:** Xin Wang, Xiao-juan Yu, Su-xia Wang, Fu-de Zhou, Ming-hui Zhao

**Affiliations:** a Renal Division, Department of Medicine, Peking University First Hospital, Beijing, China; b Peking-Tsinghua Center for Life Sciences, Beijing, China; c Institute of Nephrology, Peking University, Beijing, China; d Renal Pathology Center, Institute of Nephrology, Peking University, Beijing, China; e Key Laboratory of Renal Disease, Ministry of Health of China, Beijing, China; f Key Laboratory of CKD Prevention and Treatment, Ministry of Education of China, Beijing, China; g Research Units of Diagnosis and Treatment of Immune-Mediated Kidney Diseases, Chinese Academy of Medical Sciences, Beijing, China; h Laboratory of Electron Microscopy, Pathological Centre, Peking University First Hospital, Beijing, China

**Keywords:** LCPT, monoclonal gammopathy, free light chain, renal biopsy

## Abstract

**Background:** Light-chain proximal tubulopathy (LCPT) is a rare disease characterized by the accumulation of monoclonal light chains within proximal tubular cells. This study aimed to investigate the clinical characteristics of LCPT from a single Chinese nephrology referral center.**Methods:** Patients with kidney biopsy-proven isolated LCPT between 2016 and 2022 at Peking University First Hospital were retrospectively included. Clinical data, kidney pathological type, treatment, and prognosis were analyzed.**Results:** Nineteen patients were enrolled, the mean age at diagnosis was 57 ± 11 and the sex ratio was 6/13 (female/male). Mean proteinuria was 2.44 ± 1.89 g/24 hr and the mean estimated glomerular filtration rate (eGFR) at the point of biopsy was 59.640 ± 27.449 ml/min/1.73 m^2^. κ-restriction (84%) was dominant among LCPTs. An abnormal free light chain ratio was observed in 86% of the patients. Proximal tubulopathy with cytoplasmic inclusions accounted for the majority (53%), followed by tubulopathy associated with interstitial inflammation reaction (26%), proximal tubulopathy without cytoplasmic inclusions (16%), and proximal tubulopathy with lysosomal indigestion/constipation (5%). One patient presented with acute kidney injury and 16 patients presented with chronic kidney disease. Regarding follow-up, patients received bortezomib-based or R-CHOP chemotherapy or supportive treatment only. The mean follow-up time was 22 ± 16 months, and the mean eGFR was 63.098 ± 27.439 ml/min/1.73 m^2^ at the end of follow-up. These patients showed improved or stable kidney function.**Conclusions:** This is the first case series report of LCPT in four different pathological types in northern China. Clone-targeted chemotherapy may help preserve the kidney function in these patients.

## Introduction

LCPT was first described in 1975 and is a rare kidney disease caused by hematologic disorders with production of toxic free light chains(FLCs) [1]. LCPT is characterized by the accumulation of monoclonal light chains within proximal tubule cells (PTCs) in crystalline or non-crystalline forms, thereby causing damage to the proximal tubule [2]. In addition to multiple myeloma (MM), LCPT may be associated with monoclonal gammopathy of renal significance (MGRS), which was first introduced in 2012 by International Kidney and Monoclonal Gammopathy Research Group (IKMG) [3]. Low-molecular-weight pathogenic FLCs can be filtered through the glomerulus and then reabsorbed into PTCs by megalin/cubilin-induced endocytosis; nevertheless, these FLCs cannot undergo efficient degradation to amino acids and accumulate in PTCs instead [4].

Based on morphological manifestations, proximal tubulopathies associated with monoclonal LCs can be divided into four groups: 1) proximal tubulopathy without cytoplasmic inclusions, 2) tubulopathy associated with interstitial inflammatory reaction, 3) proximal tubulopathy with cytoplasmic inclusions, and 4) proximal tubulopathy with lysosomal indigestion/constipation [5]. Intracellular crystalline or non-crystalline accumulation of monoclonal LCs is characteristic, which results in cytoplasmic inclusions or an abundance of lysosomes in PTCs [5,6]. Furthermore, PTCs showed loss of the brush border, a common feature of LCPT. Current treatment recommendations for LCPT from the IKMG include chemotherapy or stem cell transplant to minimize the risk of end-stage kidney disease (ESKD) [7].

However, a detailed description of Chinese patients with LCPT was still lacking, especially for non-crystalline LCPT. In this study, we retrospectively included 19 Chinese patients. Clinicopathological, treatment, and follow-up data of these patients were reviewed.

## Methods

### Study population

**Figure 1.**
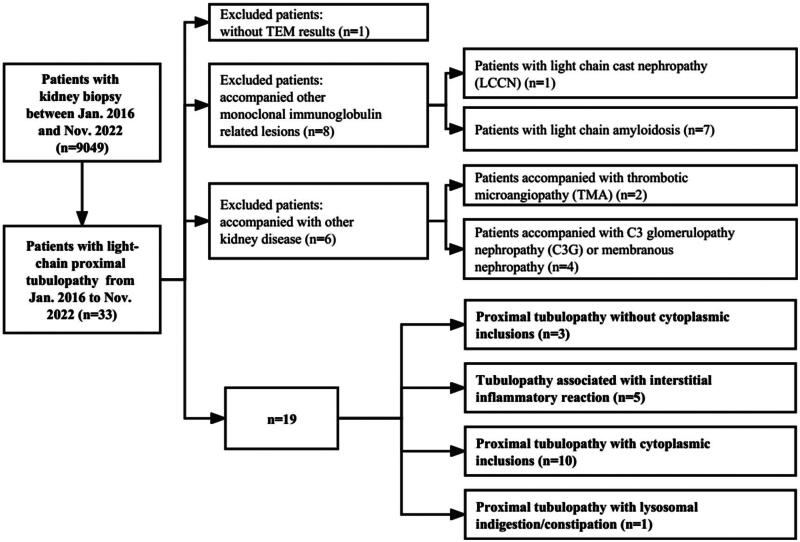
Study flow chart of patients with light-chain proximal tubulopathy (LCPT) who underwent kidney biopsies and pathological spectrum from jan. 2016 to nov. 2022.

Patients who underwent renal biopsy at Peking University First Hospital between January 2016 and November 2022 (*n* = 9049) were retrospectively reviewed. 33 patients were diagnosed with LCPT. The diagnose of LCPT required light chain-restricted staining of proximal tubular cytoplasm by immunofluorescence (IF) and the presence of intracytoplasmic proximal tubular inclusions (crystals, droplets or vacuoles) by light microscopy (LM) or transmission electron microscopy (TEM) [2]. One patient was excluded due to the absence of TEM results. Eight patients with other monoclonal immunoglobulin-related lesions were excluded, including seven with light chain amyloidosis and one with light chain cast nephropathy (LCCN). Patients with C3 glomerulopathy (C3G), thrombotic microangiopathy (TMA) and membranous nephropathy were also excluded. Nineteen patients who underwent LCPT were included in this study (Figure 1).

Written informed consent was obtained from all patients. This study was conducted in compliance with the Declaration of Helsinki and was approved by Peking University First Hospital Human Research Ethics Committee (approval number: 2017[1280]).

### Clinical, laboratory, and histopathological assessment

Clinical data, including demographic information, presenting features, medical history, and laboratory findings such as serum hemoglobin, serum creatinine, proteinuria, bone marrow biopsy test, FLC, and serum/urine immunofixation electrophoresis (IEF), were reviewed and collected through inpatient records. Patients were regularly followed-up in our outpatient clinic, and those who did not visit the clinic were contacted by phone.

Bone marrow smears and biopsies were performed to assess the hematological status. Anemia is defined as hemoglobin <130 g/L in males and <120 g/L in females according to the World Health Organization (WHO) criteria [8]. A history of hypertension is defined as a systolic blood pressure (SBP) ≥140 mmHg and/or diastolic blood pressure (DBP) ≥90 mmHg measured at the clinic [9]. The eGFR was determined using the Chronic Kidney Disease Epidemiology Collaboration (EPI) study equation. The chronic kidney disease (CKD) stage is defined according to the KIDGO guideline. The end points include death and ESKD.

### Kidney histopathology

Kidney biopsy was performed using routine immunofluorescence, light microscopy, and TEM. Fresh frozen tissue sections preserved in Zeus liquid were stained with fluorescein isothiocyanate (FITC)- labeled rabbit anti-human IgG, IgA, IgM, C1q, C3, albumin, fibrinogen, and κ and λ antibodies. IgG subclass staining was performed in patients with positive IgG staining. The results were graded from 0 to 4 according to the fluorescence intensity. Light microscopy tissue was fixed in 4% buffered formaldehyde. Consecutive serial 2 μm sections were used for histological staining, including hematoxylin and eosin (HE), Periodic acid–Schiff (PAS), periodic Schiff methenamine silver and Masson (PASM + Masson), and Masson trichrome staining. The tissue was fixed in 2.5% paraformaldehyde and embedded in Epon. Ultrathin sections were mounted on metal grids and stained with uranyl acetate. Immunogold staining for electron microscopy was used to confirm monoclonal light-chain deposition in proximal tubule cells.

*Herrera* [5] described detailed diagnostic criteria for four categories of LCPT in 2014: type I, proximal tubulopathy without cytoplasmic inclusions; type II, tubulopathy associated with interstitial inflammatory reaction; type III, proximal tubulopathy with cytoplasmic inclusions; and type IV, proximal tubulopathy with lysosomal indigestion/constipation. We categorized our patients following these criteria.

Two nephropathologists evaluated the biopsies separately, and the difference in diagnosis was resolved by re-viewing the biopsies, thus reaching a consensus.

### Statistical analysis

The statistical software SPSS 24 (IBM Corp., Armonk, NY, USA) was used. Continuous data were expressed as mean ± standard deviation (SD) for normally distributed data or median with range for non-normally distributed data. Categorical variables were presented as proportions.

## Results

### Baseline clinical characteristics

Nineteen Chinese patients with LCPT were included in this study, accounting for 0.2% of contemporaneous total kidney biopsies. The baseline demographic and clinical data are presented in Table 1.

**Table 1.** Demographic and clinical evaluation of patients with LCPT.

Characteristic	Patients(*n* = 19)
**Demographic characteristics**	
Age, y	57 ± 11
Gender	6 F/13 M
**Medical history**	
Hypertension, %	37
Diabetes, %	11
**Disease courses**	
Median duration from onset to the biopsy, m	6 (range: 2-60)
**Initial Symptoms**	
Proteinuria, %	74
Elevated serum creatinine (sCr), %	42
Fatigue, %	16
Nausea, %	11
**Serum Studies**	
Baseline sCr, μmol/L	134.91 ± 79.15
Baseline eGFR, mL/min/1.73m2	59.640 ± 27.449
Albumin, g/L	42.1 ± 8.6
Hemoglobin, g/dl	124 ± 27
**Urinary studies**	
Proteinuria (g/24 hr)	2.44 ± 1.89
N-acetyl-beta-glucosaminidase (NAG), U/L	34.8 ± 53.5
α1-microglobulin (A1M), mg/L	134.5 ± 127.8
Albumin-creatinine ratio (ACR), mg/gCr	205.67 ± 243.41
Hematuria	9
**Renal characteristics**	
CKD stage (I, II, III, IV, V)	2, 5, 7, 1, 1
Fanconi Syndrome	2
AKI	1
Acute tubular injury (ATI)	14
**Other disorders**	
Anemia	11
Hypokalemia	5
Hypophosphatemia	1
Hypocomplementemia	2

The sex ratio was 6/13 (female/male), and the mean age was 57 ± 11. Seven patients (37%) had a history of hypertension, and two (11%) had diabetes mellitus. The most common initial symptom was proteinuria (74%, 14/19), including 5 patients with elevated serum creatinine levels, 3 patients with elevated serum creatinine levels, and 2 patients with fatigue. The median duration between disease onset and renal biopsy was 6 months (range: 2 – 60 months).

The mean proteinuria was 2.44 ± 1.89 g/24 h. The mean N-acetyl-beta-glucosaminidase (NAG) was 34.8 ± 53.5 U/L (reference range: 0.3 – 12 U/L). The mean α1-microglobulin was 134.5 ± 127.8 mg/L (reference range: 0.00 − 12.00 mg/L). Of the 17 patients with urine albumin-creatinine ratio (ACR) data, 3 patients had normal ACR, whereas the other 14 patients had elevated ACR. The mean ACR was 205.67 ± 243.41 mg/gCr (range: 52.16 − 741.54 mg/gCr). Microscopic hematuria was observed in 47% (9/19) of the patients. The mean serum albumin was 42.1 ± 8.6 g/L with 5% of patients having serum albumin <30 g/L. The mean eGFR was 59.640 ± 27.449 mL/min/1.73m^2^.

Different CKD stages were frequently observed (16/19, 84%). Moreover, acute kidney injury was found in one patient. 26% patients (5/19) had hypokalemia and 5% (1/19) had hypophosphatemia. Two patients (11%) had typical Fanconi syndrome (FS), both of which belong to the crystalline LCPT. According to the WHO criteria, 11 patients (58%) had anemia. Among the 16 patients with data for serum C3 and C4 levels, 2 patients (13%) had hypocomplementemia with slightly decreased serum C3 levels as well as normal C4 levels.

### Pathological findings

The pathological data are presented in Table 2. The proportions of κ-restricted and λ-restricted LCPT patients were 84% and 16%, respectively. The IFE test revealed a monoclonal spike in serum and/or urine IFE in 16 patients. The other two patients without IFE test showed elevated serum κ FLC as well as an abnormal κ/λ ratio on serum FLC test. The remaining patient did not undergo IFE or serum FLC tests; however, this patient showed monoclonal κ-restriction expression in the plasma cells on bone marrow flow cytometry. It should be noted that serum and urine IFE were not always identical, and compressive consideration is needed.

**Table 2.** Hematological evaluation and kidney biopsy findings of patients with LCPT.

LCPT Type*	I	II	III	IV
N	3	5	10	1
**Light chain**				
κ (*n* = 16)	1	4	10	1
λ (*n* = 3)	2	1	/	/
**Paraprotein**	1 IgM λ	1 IgA λ	4 IgA κ	1 IgG κ
	1 λ only	2 IgG κ	1 IgA κ + κ	
	1 κ only	1 negative	1 IgG κ	
			1 κ only	
			1 negative	
**Urine paraprotein**	2 λ only	1 IgA λ + λ	5 κ only	1 IgG κ
	1 κ only	1 IgA κ	2 IgA κ	
		2 negatives	1 IgG κ + κ	
**Hematologic diagnoses**				
MGRS (*n* = 10)	1	3	5	1
MM (*n* = 8)	1	2	5	/
NHL (*n* = 1)	1	/	/	/
**Abnormal κ/λ ratio**	2/2	3/5	6/6	1/1
**Serum pathogenic FLC > 1000 mg/L**	2/2	3/5	4/6	0/1

* LCPT was divided into four categories according to *Herrera*^5^ as described.

Fourteen patients had serum FLC data, of whom 86% (12/14) had an abnormal κ/λ ratio (reference interval: 0.31 ∼ 1.56). 93% (13/14) of patients had the same dominant FLC type as the deposited pathogenic light chain type, and 7% (1/14) had a different dominant serum FLC type from the deposited pathogenic light chain type. However, 14% (2/14) of patients had a normal κ/λ ratio, in which one patient’s deposited FLC type was not dominant in the serum. A total of 64% (9/14) of patients had serum pathogenic FLC > 500 mg/L, as well as > 1000 mg/L.

Hematologic status showed that 53% patients (10/19) had MGRS, 42% patients (8/19) had MM and 5% patients (1/19) had non-Hodgkin lymphoma (NHL).

**Figure 2.**
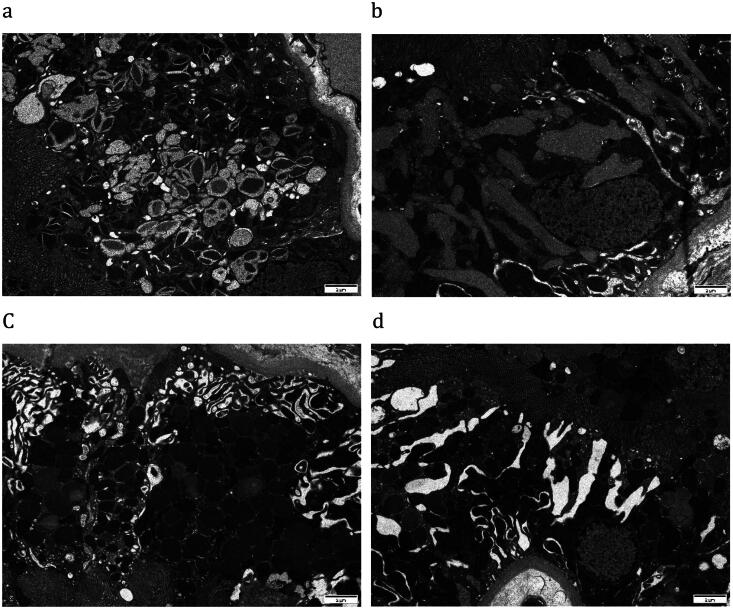
Pathologic features of LCPT by transmission electron microscopy (TEM). A) TEM showed the proximal tubular cells were occupied by crystals of varying size and shapes (×8000). B) the proximal tubular epithelial cells were variably distended by fibrillary cytoplasmic inclusions (×8000), with compression of the nuclear contours. C) The endosomes and lysosomes were increased and appeared round or ovoid which extended from the apical to the basal side of cytoplasm in a non-crystalline LCPT patient (×8000). D) PTCs of non-crystalline LCPT patients exhibited mitochondria swelling and fragmentation, which indicated the mitochondrial stress (×8000).

The LCPT categories were mainly determined using the TEM data of these 19 patients. Ten patients (53%) showed cytoplasmic inclusions in proximal tubular epithelial cells, which was consistent with proximal tubulopathy with cytoplasmic inclusions (type III). The inclusions were crystalline (Figure 2a) in eight cases and fibrillary (Figure 2b) in one. The other 9 patients (47%) did not exhibit cytoplasmic inclusions, of which 3 (16%) were identified as proximal tubulopathy without cytoplasmic inclusions (Type I), 5 (26%) were identified as tubulopathy associated with interstitial inflammatory reaction (Type II), and 1 (5%) was identified as proximal tubulopathy with lysosomal indigestion/constipation (Type IV). An abundance of lysosomes could be found in PTCs of type I, type II, and type IV (Figure 2c), which may indicate substrate overload within lysosomes. Mitochondrial swelling and fragmentation were commonly observed in these LCPT cases, suggesting mitochondrial stress (Figure 2d).

## Treatment and follow-up

Follow-up data were available for 8 patients with LCPT, which are presented in Table 3. In terms of follow-up, six patients received bortezomib-based chemotherapy, one patient received R-CHOP chemotherapy, another one patient only received supportive treatment. The mean follow-up period was 22 ± 16 months. Two patients in the chemotherapy group had improved kidney function, and the other 6 patients had stable kidney function. Two patients who received chemotherapy received autologous stem cell transplant (ASCT). Chemotherapy included various combinations of dexamethasone, bortezomib, cyclophosphamide, lenalidomide, doxorubicin, vincristine, daratumumab, and rituximab. None of these 8 subjects died or presented with ESKD until the end of the follow-up period.

**Table 3.** Chemotherapeutic strategy and outcomes of LCPT patients.

LCPT Type	Hematologic diagnoses	Therapeutic agent	SCT	sCr (initial)	eGFR (initial)	sCr (final)	eGFR (final)	Hematologic outcomes	Follow-up time (m)
I	NHL	R-CHOP	No	48.59	98.767	43.9	101.406	SD	5
II	MGRS	Supportive	No	71.33	97.537	/	/	Un	38
	MM	BCD	Yes	68.6	101.119	83.6	88.985	sCR	17
III	MM	BCD	No	131.34	49.065	177	33.967	SD	53
	MM	BCD + BR	No	99.3	48.678	121.6	37.571	SD	22
	MGRS	BCD	No	137	46.953	/	/	Un	15
	MM	BCD + DVD	No	132.3	45.977	99.16	64.697	VGPR	15
	MM	BCD	Yes	193.7	33.607	134.3	51.961	CR	9

BCD: bortezomib + cyclophosphamide + dexamethasone; BR: lenalidomide + bortezomib; DVD: daratumumab + bortezomib + dexamethasone; R-CHOP, rituximab + cyclophosphamide + doxorubicin + vincristine + prednisone.

CR, complete remission; sCR, stringent complete response; VGPR, very good partial response; SD, stable hematologic disease; Un: Unknown.

## Discussion

LCPT is an extremely rare disease, accounting for only 4% − 5% of monoclonal light chain-associated kidney diseases [2,10]. Monoclonal FLCs are small molecular weight proteins that can be freely filtered through the glomerular filtration barrier. The filtered FLCs can be reabsorbed by the PTCs through the megalin/cubilin complex [4]. Normally, FLCs are degraded into amino acids for recycling. However, toxic LCPT-inducing FLCs cannot be properly digested, leading to cytoplasmic storage. The toxicity of FLC and the resultant different pathologic types were speculated to be due to special amino acid mutations in the variable domain of FLC, resulting in changes in biochemical characteristics [11–13].

The diagnosis of crystalline LCPT can be made by TEM showing crystals with different shapes in the cytoplasm of proximal tubular epithelial cells with immuno-EM to confirm κ light chain restriction deposition, and light microscopy can show inclusions in the cytoplasm on trichrome staining. Aside from proximal tubular epitheliums, κ light chain crystals could be found in glomerular podocytes, parietal epithelial cells, and interstitial histocytes, but proximal tubular epithelial cells were predominant. Proximal tubulopathy with lysosomal indigestion/constipation was observed by light microscopy, showing numerous enlarged lysosomes gathered in the cytoplasm on trichrome staining, and lysosomes were PAS-negative. On immuno-EM, the increased lysosomes showed κ or λ light chain restriction deposition.

Proximal tubulopathy without cytoplasmic inclusions showed ATI or necrosis without crystals or lysosomal constipation. Tubulopathy associated with an interstitial inflammatory reaction showed interstitial inflammation and ATI without crystals on EM or lysosome constipation on light microscopy. EM data showed abnormal lysosomes with κ or λ light-chain restriction deposition in both proximal tubulopathy without cytoplasmic inclusions and tubulopathy associated with interstitial inflammatory reaction.

Thus, transmission electron microscopy and immuno-EM are essential tools for accurately confirming crystalline LCPT diagnosis and for the differential diagnosis of different pathologic types of LCPT. The immunogold electron microscopy approach could provide precise subcellular localization of the light chain to determine the pathogenic type because, in some situations, pathogenic FLC may not be the dominant type in the serum.

All the reported crystalline LCPT cases, including our patients, were caused by the κ light chain. For non-crystalline LCPT, the pathogenic FLC type can be either κ or λ. In this study, 2/3 of the non-crystalline LCPT patients were κ-restricted, and 1/3 patient were λ-restricted. Complete or partial FS may occur on LCPT patients. In this study, 10% of the patients (2/19) exhibited FS, and they were all crystalline LCPT patients, which was relatively lower than reported series before [14]. This indicates that the FS-inducing mechanism may be independent of the crystal-forming process. Symptoms of hypophosphatemia, including osteomalacia and myoasthenia, or hypokalemia, including paralysis or fatigue, are the most important. Laboratory tests have confirmed impaired proximal tubular function, including amino aciduria, glycosuria, hyperphosphatemia, and type II tubular acidosis [15].

LCPT can also occur in combination with other monoclonal immunoglobulin-related kidney diseases. In addition, the symptoms and laboratory tests of LCPT can be overshadowed and misdiagnosed on kidney biopsies, especially in non-crystalline LCPT cases, because more evidence is needed to distinguish it from other diseases.

The treatment of LCPT is based on eradicating light chain secreting plasma cells or B cells by bortezomib-based chemotherapy or stem cell transplantation. For malignant patients, such as those with multiple myeloma, treatment is based on hematologic diseases. For MGRS patients, cyclophosphamide-, bortezomib-, or thalidomide-based chemotherapy is usually recommended for patients with crystalline LCPT to improve proximal tubular function and preserve kidney function [7]. Crystalline LCPT patients had at least stable hematologic disease at last follow-up, and more than one third of them received bortezomib [2]. Anti-CD38 monoclonal antibody (daratumumab) has also been used in crystalline LCPT, showing promising results [16]. In our study, five MGRS-crystalline LCPT patients who received bortezomib-based chemotherapy with 2/5 showed hematological response (CR + VGPR + PR), and two patients did not show improved eGFR. One patient received daratumumab plus bortezomib-based chemotherapy with hematologic VGPR response.

Chemotherapy is logical in patients with proximal tubulopathy without cytoplasmic inclusions, and tubulopathy associated with interstitial inflammatory reaction. However, these two pathological types were not widely recognized by pathologists, hematologists, and nephrologists as crystalline LCPT, and these patients might have been undertreated, as shown in our study. Patients with proximal tubulopathy with lysosomal indigestion/constipation usually have very mild clinical proximal tubular dysfunction and preserved kidney function, and the necessity for chemotherapy must be evaluated for each patient to balance the benefits and risks. Two patients of these two types received chemotherapy in our study.

The limitations of this study include its small sample size, retrospective design, and various treatments. A prospective larger sample of LCPT patients is needed for further clinical, treatment, and long-term follow-up analyses.

## Conclusion

The four patients with different pathological LCPT had different clinical manifestations and indications for chemotherapy. The kidney outcome of patients with LCPT treated with chemotherapy targeting aberrant plasma clones was effective in improving proximal tubular function and eGFR.
